# Systematic genetic testing for recessively inherited monogenic diabetes: a cross-sectional study in paediatric diabetes clinics

**DOI:** 10.1007/s00125-021-05597-y

**Published:** 2021-10-23

**Authors:** Kashyap A. Patel, Mehmet N. Ozbek, Melek Yildiz, Tulay Guran, Cemil Kocyigit, Sezer Acar, Zeynep Siklar, Muge Atar, Kevin Colclough, Jayne Houghton, Matthew B. Johnson, Sian Ellard, Sarah E. Flanagan, Filiz Cizmecioglu, Merih Berberoglu, Korcan Demir, Gonul Catli, Serpil Bas, Teoman Akcay, Huseyin Demirbilek, Michael N. Weedon, Andrew T. Hattersley

**Affiliations:** 1 Institute of Biomedical and Clinical Science, College of Medicine and Health, University of Exeter, Exeter, UK; 2 Department of Paediatric Endocrinology, Gazi Yasargil Diyarbakir Training and Research Hospital, Diyarbakir, Turkey; 3 Department of Paediatric Endocrinology, Kanuni Sultan Suleyman Training and Research Hospital, Istanbul, Turkey; 4 Department of Paediatric Endocrinology, Istanbul Faculty of Medicine, Istanbul University, Istanbul, Turkey; 5 Department of Paediatric Endocrinology and Diabetes, Marmara University Hospital, Istanbul, Turkey; 6 Department of Paediatric Endocrinology, Tepecik Training and Research Hospital, Izmir, Turkey; 7 Department of Paediatric Endocrinology, Dokuz Eylul University, Izmir, Turkey; 8 Division of Paediatric Endocrinology, Dr Behcet Uz Child Disease and Paediatric Surgery Training and Research Hospital, Izmir, Turkey; 9 Department of Paediatric Endocrinology, Ankara University School of Medicine, Ankara, Turkey; 10 Department of Paediatric Endocrinology, Kocaeil University Hospital, Izmit, Turkey; 11 Department of Paediatric Endocrinology, Suleyman Demirel University, Isparta, Turkey; 12 Department of Molecular Genetics, Royal Devon and Exeter National Health Service Foundation Trust, Exeter, UK; 13 Department of Paediatric Endocrinology, Istinye University, Gaziosmanpasa Medical Park Hospital, Istanbul, Turkey; 14 Department of Paediatric Endocrinology, Hacettepe University Faculty of Medicine, Ankara, Turkey

**Keywords:** Diabetes syndrome, MODY, Monogenic diabetes, Recessive monogenic diabetes, Syndromic diabetes, Type 1 diabetes, Type 1 diabetes genetic risk score

## Abstract

**Aims/hypothesis:**

Current clinical guidelines for childhood-onset monogenic diabetes outside infancy are mainly focused on identifying and testing for dominantly inherited, predominantly MODY genes. There are no systematic studies of the recessively inherited causes of monogenic diabetes that are likely to be more common in populations with high rates of consanguinity. We aimed to determine the contribution of recessive causes of monogenic diabetes in paediatric diabetes clinics and to identify clinical criteria by which to select individuals for recessive monogenic diabetes testing.

**Methods:**

We conducted a cross-sectional study of 1093 children from seven paediatric diabetes clinics across Turkey (a population with high rates of consanguinity). We undertook genetic testing of 50 known dominant and recessive causes of monogenic diabetes for 236 children at low risk of type 1 diabetes. As a comparison, we used monogenic diabetes cases from UK paediatric diabetes clinics (a population with low rates of consanguinity).

**Results:**

Thirty-four children in the Turkish cohort had monogenic diabetes, equating to a minimal prevalence of 3.1%, similar to that in the UK cohort (*p* = 0.40). Forty-one per cent (14/34) had autosomal recessive causes in contrast to 1.6% (2/122) in the UK monogenic diabetes cohort (*p* < 0.0001). All conventional criteria for identifying monogenic diabetes (parental diabetes, not requiring insulin treatment, HbA_1c_ ≤ 58 mmol/mol [≤7.5%] and a composite clinical probability of MODY >10%) assisted the identification of the dominant (all *p* ≤ 0.0003) but not recessive cases (all *p ≥* 0.2) in Turkey. The presence of certain non-autoimmune extra-pancreatic features greatly assisted the identification of recessive (*p* < 0.0001, OR 66.9) but not dominant cases.

**Conclusions/interpretation:**

Recessively inherited mutations are a common cause of monogenic diabetes in populations with high rates of consanguinity. Present MODY-focused genetic testing strategies do not identify affected individuals. To detect all cases of monogenic paediatric diabetes, it is crucial that recessive genes are included in genetic panels and that children are selected for testing if they have certain non-autoimmune extra-pancreatic features in addition to current criteria.

**Graphical abstract:**

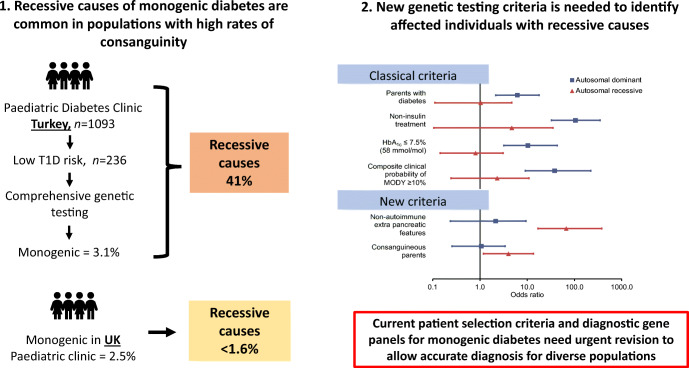

**Supplementary Information:**

The online version of this article (10.1007/s00125-021-05597-y) contains peer-reviewed but unedited supplementary material.

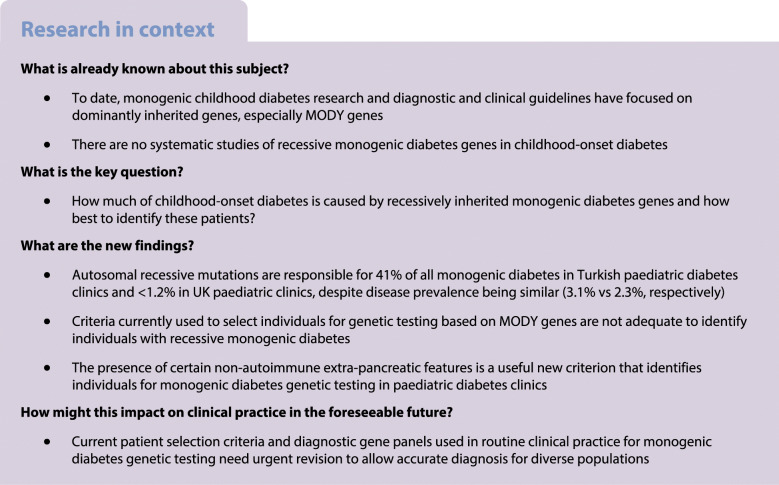



## Introduction

Research into childhood-onset monogenic diabetes outside the neonatal period has focused on dominantly inherited genes, especially MODY genes (*HNF1A*, *HNF4A*, *GCK*). Clinical studies of MODY and other dominantly inherited monogenic diabetes genes in children [1] have influenced clinical practice and international guidelines towards patient selection criteria for identifying dominantly inherited causes [2].

Mutations in recessively inherited genes are an important cause of monogenic diabetes outside infancy. However, there have been no systematic studies of recessive monogenic diabetes genes in childhood-onset diabetes in populations with high rates of consanguinity. If these genes are common in certain populations, such studies are needed to improve patient selection criteria, gene panels and clinical guidelines for childhood-onset diabetes [1, 2].

In this study, we undertook systematic testing for dominant and recessive monogenic diabetes genes in paediatric diabetes clinic attendees in Turkey (a population with high rate of consanguinity [~20%] [3]) and the UK (a population with low rate of consanguinity [<1%] [4]). We assessed the contribution of recessively inherited mutations to monogenic diabetes in childhood-onset diabetes and identified a new clinical criterion to use in routine clinical practice to select individuals for recessive monogenic diabetes genetic testing.

## Methods

### Study population

We conducted a cross-sectional study of 1093 children attending paediatric diabetes clinics in seven hospitals across Turkey, recruited from April 2016 to May 2017 (ESM Fig. 1). Individuals with diabetes onset between 6 months and 20 years of age were eligible for the study. Baseline HbA_1c_, C-peptide and islet autoantibody status were recorded during routine clinical care. The presence of non-autoimmune extra-pancreatic features (i.e. in addition to diabetes), such as deafness, developmental delay or anaemia, was recorded.

We collected saliva (1 ml) from which DNA was extracted at LGC genomics (Hoddesdon, UK). The characteristics of the cohort are provided in ESM Table 1. The self-reported rate of parental consanguinity (second cousins or closer relatives) was 20%. The sample size calculation is provided in ESM Methods. The study was approved by the Kocaeli University ethics committee (reference KOU KAEK 2016/01) and was conducted in accordance with the Declaration of Helsinki. All participants or their parents gave informed consent.

For a comparison population with a low rate of consanguinity, we included two cohorts of children with monogenic diabetes from the UK (all screened using the same comprehensive genetic testing as the current study): one from our previous systematic study in the same setting (*n* = 20) [5] and one comprising routine UK diagnostic referrals to our Exeter genetics laboratory with the same age at diagnosis (0.5–20 years, *n* = 102).

### Genetic data

#### Type 1 diabetes genetic risk score

We used 30 type 1 diabetes-associated SNPs to generate a weighted type 1 diabetes genetic risk score (T1D-GRS), as previously described [6] (ESM Methods, ESM Table 2). T1D-GRS centiles were derived from 1963 gold-standard European type 1 diabetes patients from the Wellcome Trust Case Control Consortium (WTCCC) [7]. The T1D-GRS of the 472 Turkish children from our cohort with definite type 1 diabetes (clinically diagnosed, insulin treated, islet autoantibody-positive) had a similar median and distribution to those of the 1963 European children (median [IQR] 0.279 [0.260–0.298] vs 0.280 [0.262–0.298], Mann–Whitney *U* test *p* = 0.48, two-sample Kolmogorov–Smirnov test *p* = 0.37) (ESM Fig. 2). These data support our use of T1D-GRS centiles from the European cohort to categorise Turkish children in the previously described low, moderate and high type 1 diabetes genetic risk groups [6, 8, 9]. T1D-GRS was defined as follows: low, GRS <0.234 (<fifth centile of the WTCCC cohort); moderate, GRS 0.234–0.280 (fifth to 50th centile of the WTCCC cohort); and high, GRS >0.280 (>50th centile of the WTCCC cohort).

#### Genetic testing for monogenic diabetes

Based on our previous work [6, 9], we used a combination of T1D-GRS and islet autoantibody status to select 236/1093 children with high to moderate likelihood of monogenic diabetes (i.e. low/moderate risk of type 1 diabetes) for genetic testing (ESM Fig. 1): 111 children with low T1D-GRS irrespective of islet autoantibody status and 125 islet-autoantibody-negative children with a moderate T1D-GRS (ESM Fig. 1). Genetic testing was not carried out for 857 individuals with a low likelihood of monogenic diabetes (high T1D-GRS [*n* = 516], moderate T1D-GRS and either islet autoantibody-positive [*n* = 208] or missing autoantibody status [*n* = 133] [ESM Fig. 1]). This allowed us to select individuals for genetic testing independent of the clinical phenotype with high to moderate probability of monogenic diabetes. We used targeted next generation sequencing to analyse all known dominant and recessive genes causing monogenic diabetes (*n* = 50) (ESM Methods and ESM Table 3).

### Statistical analysis

Data were analysed using STATA16 (StataCorp, USA). The Mann–Whitney *U* test and the Fisher Exact test were used to compare continuous and categorical variables, respectively. The distribution of T1D-GRS between groups was compared using a two-sample Kolmogorov–Smirnov test. We used the exact binomial CI (Clopper–Pearson) method to calculate a 95% CI for the proportion. The prevalence of monogenic diabetes was compared between the study cohorts using the two-sample test for equality of proportions. We assessed the strength of any association between a given clinical feature and the presence of monogenic diabetes by calculating the OR, its exact 95% CI and two-tailed Fisher’s exact *p* value. WHO child growth BMI centiles were generated using the Zanthro package [10], and the clinical probability of MODY was determined using our validated statistical model [11].

## Results

### Recessive monogenic diabetes causes

**Fig. 1:**
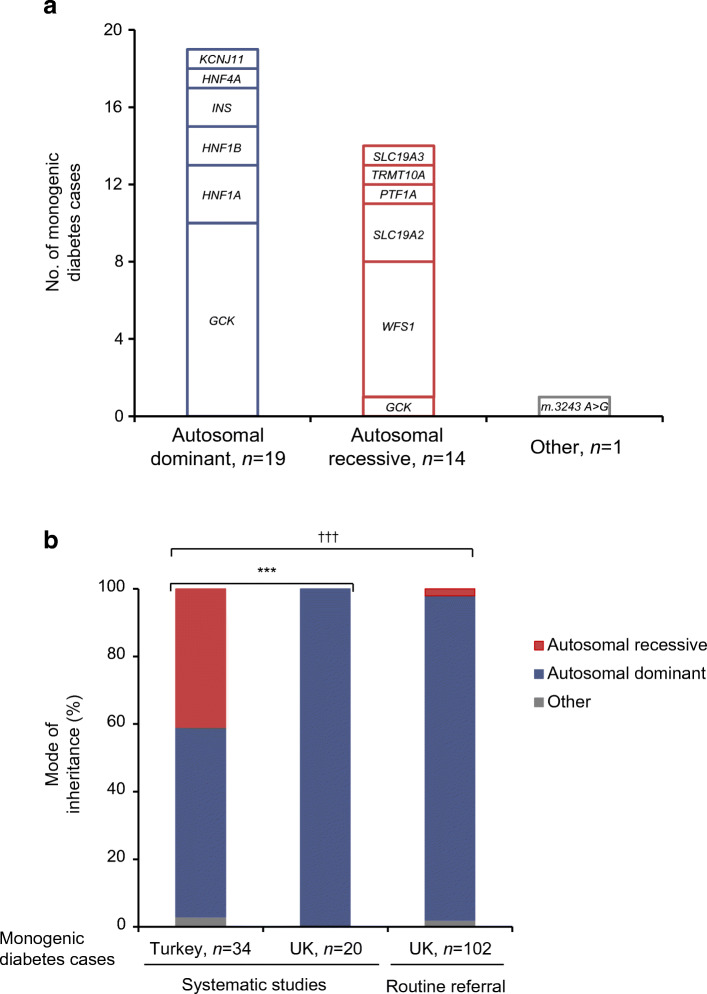
Recessive and dominant inherited causes of monogenic diabetes in the Turkish and UK populations. (**a**) Bar chart showing the number of individuals with monogenic diabetes by gene symbol and mode of inheritance (*n* = 34) identified in Turkish paediatric clinics (age at diagnosis 0.5–20 years). All monoallelic variants were included under autosomal dominant inheritance, and all biallelic variants were included under autosomal recessive inheritance. ‘Other’ includes mitochondrial variants. (**b**) Comparison of the mode of inheritance of monogenic diabetes in individuals identified from the current study in Turkey (a country with ~20% consanguinity) and the UK (a country with <1% consanguinity). Data from the UK are from individuals with monogenic diabetes identified in our previous systematic study in the same setting (paediatric diabetes clinic, *n* = 20/808) (11) as well as cases identified from routine diagnostic referrals diagnosed between 0.5 and 20 years (*n* = 102). All cases were identified by the same comprehensive genetic testing of all known autosomal dominant and recessive causes of monogenic diabetes as the current study. ***p = 0.0005 for the current study vs previous systematic study from the UK; ^†††^*p* = 1 × 10^−8^ for the current study vs routine diagnostic referral from the UK

Genetic analysis identified 34 individuals with monogenic diabetes in the Turkish cohort, equating to a minimum prevalence of 3.1% (34/1093; 95% CI 2.2, 4.3) (Fig. 1a and ESM Table 4), similar to that in the UK cohorts (2.5% [95% CI 1.5, 3.8], *p* = 0.40) [5], although genetic causes differed markedly.

Autosomal dominant causes (monoallelic pathogenic variants) were less common in the Turkish cohort than in the UK cohorts (56% vs 100% [systematic study in UK paediatric clinic, *p* = 0.64] and 96% [routine UK referral cohort, *p* = 1 × 10^−7^]). Autosomal recessive causes (biallelic pathogenic variants) accounted for 14 Turkish individuals (41%) but were rare in the UK: 0/20 in systematic study, *p* = 7 × 10^−4^ [5] and 2/102 in routine UK referrals, *p* = 2 × 10^−8^ (Fig. 1b and ESM Tables 5, 6).

### Performance of selection criteria commonly used for genetic testing

**Fig. 2:**
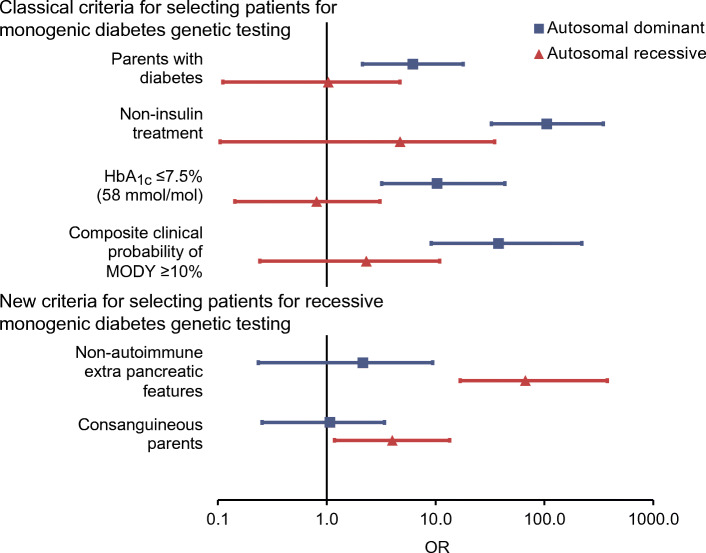
Performance of selection criteria for identifying monogenic diabetes in Turkish paediatric diabetes clinics. OR and 95% CI is shown for each criterion for autosomal dominant and autosomal recessive cases separately

The clinical characteristics of the Turkish children differed for those with dominant vs recessive causes of type 1 diabetes and when comparing these children with the remainder of the study population (ESM Table 7). Similar to the UK population, non-insulin treatment, a parent with diabetes, HbA_1c_ ≤ 58 mmol/mol (7.5%) and a composite clinical probability of MODY ≥10% were useful in identifying autosomal dominant diabetes in the Turkish cohort (OR 105.1, 6.2, 10.3 and 38, respectively, all *p ≤* 0.0003) (Fig. 2 and ESM Table 8). However, none of these criteria identified autosomal recessive diabetes (all *p ≥* 0.2).

### New criteria for selecting individuals for recessive monogenic diabetes testing

The presence of certain non-autoimmune extra-pancreatic features markedly improved the identification of monogenic diabetes with autosomal recessive cause when compared with the rest of the cohort (OR 66.9 [95% CI 16.8, 379.1], *p* = 6× 10^−12^) (Fig. 2 and ESM Table 8). Eighteen discrete non-autoimmune extra-pancreatic features were reported in individuals with monogenic diabetes (ESM Table 9); deafness, anaemia and developmental delay were seen in 9/14 autosomal recessive cases (ESM Table 4). Consanguinity of parents also pointed towards autosomal recessive diabetes to a lesser degree (OR 4 [95% CI 1.2, 13.5], *p* = 0.01). However, neither criteria identified autosomal dominant diabetes (all *p* > 0.2) (Fig. 2 and ESM Table 8).

## Discussion

We show that mutations in recessive genes are major contributors to childhood-onset monogenic diabetes in a population with a high rate of consanguinity. Comprehensive genetic testing (of both dominant and recessive genes) shows for the first time that despite having a similar overall prevalence in Turkish and the UK populations, mutations in recessive monogenic diabetes genes were 40-fold more common in Turkey and accounted for nearly half of the cases. Although this was expected due to the high rate of consanguinity, the extent to which recessive aetiology contributes overall is not known. Previous studies in this population mainly focused on dominant genes [12–15], and currently available gene panel tests for monogenic diabetes lack recessive genes (ESM Table 10). Of the 34 gene panels for MODY/monogenic diabetes listed in the NCBI Gene Testing Registry, only six panels and one panel included *WFS1* and *SLC19A2*, respectively (the two commonest causes in our Turkish cohort) (ESM Table 10).

Genetic testing independent of clinical features showed that current criteria (derived predominantly from studies of MODY genes in European ancestry populations) were still useful for identifying MODY in Turkish children, as previously reported [12, 14, 15]. However, these criteria did not identify recessive cases. We show that the presence of certain non-autoimmune extra-pancreatic features is highly suggestive of autosomal recessive causes (OR 66.9). It is known that most recessive genetic subtypes have additional features, but genetic testing is often restricted to individuals with features suggestive of the syndrome. In our study, only 2/19 individuals were clinically suspected of having a recessive cause (one *WFS1* and one *SLC19A2*), whereas the rest had atypical features (ESM Table 4). These data suggest that the presence of additional non-autoimmune features would be a useful addition to current criteria for selecting individuals for monogenic diabetes genetic testing. This simple recommendation is easy to implement and should be incorporated into guidelines worldwide. This should be followed by urgent improvement of current gene panels, which lack recessive genes.

We strongly recommend the inclusion of recessive genes in the monogenic diabetes panel, even in countries with low rates of consanguinity. It is not practical to have different gene panels for monogenic diabetes within a single country. It is sensible that all patients are tested for recessive causes and this would avoid any hesitancy that healthcare professionals in countries with low rates of consanguinity might have in enquiring about consanguinity of parents. There is a small additional cost of adding the recessive genes with next generation sequencing methods, which are now widely used.

A limitation of our study is that we did not genetically test children at high diabetes genetic risk who were islet autoantibody negative. However, we expect that testing of these children would identify only one or two additional cases of monogenic diabetes. Since we did not select children based on clinical phenotype, these additional cases would not alter the proportions of recessive and dominant cases and thus would not change the overall conclusion of the study. However, this additional testing may result in a slightly higher prevalence of monogenic diabetes (3.3% instead of the present minimum prevalence of 3.1%).

In conclusion, mutations in recessive monogenic diabetes genes are an important contribution to childhood-onset monogenic diabetes outside infancy in populations with high rates of consanguinity. Our results highlight the need for modifying patient selection criteria for monogenic diabetes genetic testing and suggest the urgent need to include recessive aetiologies in the current diagnostic gene panels to allow accurate testing of diverse populations.

## Supplementary information

ESM 1 (PDF 504 kb)

## Data Availability

The datasets supporting the current study have not been deposited in a public repository due to institutional ethics restrictions but are available from the corresponding author on request.

## Abbreviations

T1D-GRS: Type 1 diabetes genetic risk score
WTCCC: Wellcome Trust Case Control Consortium
