# Supplementary material for: Systematic genetic testing for recessively inherited monogenic diabetes: a cross-sectional study in paediatric diabetes clinics
Source: Diabetologia. 2021 Oct 23;65(2):336–42. doi: 10.1007/s00125-021-05597-y (PMC8741690; doi:10.1007/s00125-021-05597-y)
Supplement: Supplementary file 1 — (PDF 504 kb) [file 125_2021_5597_MOESM1_ESM.pdf]

ESM Supplemental Methods

Sample size: Our study aims to determine the prevalence of monogenic diabetes in Turkish paediatric clinics. Therefore, we have used a precision-based sample size calculation to assess if we had the power to detect a similar prevalence to that found in a previous UK paediatric study [1]. The sample size based on the previous systematic study of monogenic diabetes in paediatric clinics in the UK which identified 20 patients with monogenic diabetes from total 808 children (2.5%). Based on this, to identify at least 20 patients at 2.5% prevalence at 90% power, we needed a sample size of 1034. We recruited 1093 participants. This provided 90% power to detect prevalence of monogenic diabetes of 2.5% with a 90% confidence interval of 1.6% to 3.6%.

Type 1 diabetes genetic risk score

We used 30 type 1 diabetes (T1D) associated single nucleotide polymorphisms (SNPs) to generate the weighted T1D genetic risk score (T1D-GRS) as previously described [2]. The list of 30 SNPs along with weights used in the generation of risk score is provided below. SNPs were genotyped using fluorescence-based competitive-allele specific assay at LGC Genomics, Hoddesdon, UK. We were unable to generate T1D-GRS in 34 individuals in whom either genotyping results were missing for one of the alleles that have the greatest influence on the GRS (DR3/DR4-DQ8 or HLA-DRB1 15), or who were missing more than two other SNPs. Individuals with missing GRS were excluded from the final study cohort.

Genetic testing

The coding regions and 50 nucleotides of flanking intronic sequence of 50 known monogenic diabetes genes were analysed by targeted-next generation sequencing (t-NGS) according to the methodology described by Ellard et al.[3] The panel includes genes known to cause neonatal diabetes, MODY, type 1 diabetes as part of an autoimmune syndrome, lipodystrophy, severe insulin resistance, other forms of syndromic diabetes and the mitochondrial mutation m.3243A>G associated with maternally inherited diabetes and deafness. The pathogenicity of variants was determined using the five-tier classification system as per the American College of Medical Genetics (ACMG) guidelines [4].

ESM Table 1. Characteristics of the study cohort

	Whole cohort, n=1093
Age at diagnosis (years)	8.1 (5-11.5), 1093
Duration at recruitment (years)	3.1 (1.2-5.6), 1093
Age at recruitment (years)	12.5 (9.1-15.4), 1093
BMI centile	61 (33-82), 1092
BMI centile ≥95th	86/1092 (8)
Female	547/1093 (50)
Extra pancreatic features	
Autoimmune	82/1093 (8)
Non-Autoimmune	68/1093 (6)
Parents with diabetes	156/1076 (15)
Consanguineous parents	223/1093 (20)
Any antibody positive	479/730 (66)
Duration at antibody measured, months	0 (0-0), 682
C-peptide within 6 months from diagnosis (pmol/l)	156 (83-283), 603
HbA1c at recruitment, %	8.7 (7.5-10.3), 1076
HbA1c at recruitment, mmol/mol	72 (58-89), 1076
Treatment	
None	21/1093 (2)
OHA	9/1093 (1)
Insulin	1044/1093 (96)
Insulin + OHA	19/1093 (2)
Insulin dose (units/kg/day)	0.9 (0.7-1.1), 996
T1D-GRS	0.278 (0.257-0.297), 1093
T1D-GRS categories	
T1D-GRS <5th centile (<0.234)	111/1093 (10)
T1D-GRS 5th-25th centile (0.234-0.262)	224/1093 (20)
T1D-GRS 25th-50th centile (0.263-0.280)	242/1093 (22)
T1D-GRS >50th centile (>0.280	516/1093 (47)
Composite clinical probability of MODY ≥10%	76/915 (8)

Data are median (IQR) for continuous variable or number (%) for categorical variables. Number of people where the data was available is indicated after comma for continuous variables and after forward slash for categorical variables. T1D-GRS centile were based on 1963 European gold standard T1D population from WTCCC case control consortium study. OHA, Oral hypoglycaemic agents; T1D-GRS, Type 1 diabetes – Genetic Risk Score; MODY, Maturity Onset Diabetes of the Young.

ESM Table 2. diabetes single nucleotide polymorphisms (SNPs) and their weights included in the generation of genetic risk score. Effect allele is the risk increasing allele on the positive strand. The rs7454108 genotypes (+ve strand) corresponding to the different DR3/DR4 genotypes are: TT/TT = DR3/DR3; TC/CT = DR3/DR4; TC/TT = DR3/X; CC/CC = DR4/DR4; CC/CT = X/DR4; CC/TT = X/X.

SNP	Gene	Odds Ratio	Weight	Effect Allele
rs2187668, rs7454108	*DR3/DR4-DQ8*	48.18	3.87	
	DR3/DR3	21.12	3.05	
	DR4-DQ8/DR4-DQ8	21.98	3.09	
	DR4-DQ8/X	7.03	1.95	
	DR3/X	4.53	1.51	
rs1264813	*HLA_A_24*	1.54	0.43	T
rs2395029	*HLA_B_5701*	2.5	0.92	T
rs3129889	*HLA_DRB1_15*	14.88	2.70	A
rs2476601	*PTPN22*	1.96	0.67	A
rs689	*INS*	1.75	0.56	T
rs12722495	*IL2RA*	1.58	0.46	T
rs2292239	*ERBB3*	1.35	0.30	T
rs10509540	*C10orf59*	1.33	0.29	T
rs4948088	*COBL*	1.3	0.26	C
rs7202877		1.28	0.25	G
rs12708716	*CLEC16A*	1.23	0.21	A
rs3087243	*CTLA4*	1.22	0.20	G
rs1893217	*PTPN2*	1.2	0.18	G
rs11594656	*IL2RA*	1.19	0.17	T
rs3024505	*IL10*	1.19	0.17	G
rs9388489	*C6orf173*	1.17	0.16	G
rs1465788		1.16	0.15	C
rs1990760	*IFIH1*	1.16	0.15	T
rs3825932	*CTSH*	1.16	0.15	C
rs425105		1.16	0.15	T
rs763361	*CD226*	1.16	0.15	T
rs4788084	*IL27*	1.16	0.15	C
rs17574546		1.14	0.13	C
rs11755527	*BACH2*	1.13	0.12	G
rs3788013	*UBASH3A*	1.13	0.12	A
rs2069762	*IL2*	1.12	0.11	A
rs2281808		1.11	0.10	C
rs5753037		1.1	0.10	T

ESM Table 3. The monogenic diabetes genes analysed in the study.

Gene	Genbank Reference Sequence	Phenotype	Inheritance
ABCC8	NM_001287174	Permanent neonatal diabetes	Dominant (often de novo) or recessive
		Transient neonatal diabetes	Dominant (often de novo) or recessive
		MODY	Dominant
AGPAT2	NM_006412	Congenital generalised lipodystrophy	Recessive
AKT2	NM_001626	Lipodystrophy and severe insulin resistance	Dominant
APPL1	NM_012096	MODY	Dominant
BSCL2	NM_032667	Congenital generalised lipodystrophy, severe insulin resistance and diabetes	Recessive
CTLA4	NM_005214	Type V autoimmune lymphoproliferative syndrome and autoimmune diabetes	Dominant
CEL	NM_001807	MODY	Dominant
CISD2	NM_001008388	Wolfram Syndrome 2 (diabetes mellitus, hearing loss, optic atrophy and defective platelet aggregation).	Recessive
COQ2	NM_015697	Coenzyme Q10 deficiency, primary, 1 (hyperglycaemia reported)	Recessive
EIF2AK3	NM_004836	Wolcott-Rallison syndrome	Recessive
FOXP3	NM_014009	Immunodysregulation, polyendocrinopathy, and enteropathy, X-linked syndrome (IPEX)	X-Linked Recessive
GATA4	NM_002052	Permanent neonatal diabetes with pancreatic agenesis and congenital heart defects	Dominant (often de novo)
GATA6	NM_005257	Permanent neonatal diabetes with pancreatic agenesis and congenital heart defects	Dominant (often de novo)
GCK	NM_000162	Permanent neonatal diabetes	Recessive
		MODY	Dominant
GLIS3	NM_001042413	Permanent neonatal diabetes with congenital hypothyroidism	Recessive
HNF1A	NM_000545	MODY	Dominant
HNF1B	NM_000458	Renal Cysts and Diabetes syndrome (RCAD)	Dominant (often de novo)
HNF4A	NM_175914	MODY	Dominant
IER3IP1	NM_016097	microcephaly, epilepsy, and diabetes syndrome (MEDS)	Recessive
IL2RA	NM_000417	Immunodeficiency 41 with lymphoproliferation, autoimmunity and autoimmune diabetes	Recessive
INS	NM_001185098	Permanent neonatal diabetes	Dominant

			(often de novo) or recessive
		Transient neonatal diabetes	Dominant (often de novo) or recessive
		MODY	Dominant
INSR	NM_000208	Severe insulin resistance	Dominant
ITCH	NM_001257138	Multisystem autoimmune disease with facial dysmorphism and autoimmune diabetes	Recessive
KCNJ11	NM_000525	Permanent neonatal diabetes	Dominant (often de novo)
		Transient neonatal diabetes	Dominant (often de novo)
		MODY	Dominant
LMNA	NM_170707	Familial Partial Lipodystrophy (FPLD2) and insulin resistance	Dominant
LRBA	NM_001199282	Immunodysregulation and autoimmune diabetes	Recessive
MNX1	NM_005515	Neonatal diabetes & IUGR	Recessive
MTTL1 g.3243A>G	NC_012920	Maternally inherited diabetes and deafness (MIDD)	Mitochondrial
NEUROD1	NM_002500	Permanent neonatal diabetes and neurological abnormalities	Recessive
		MODY	Dominant
NEUROG3	NM_020999	Permanent neonatal diabetes with congenital malabsorptive diarrhoea	Recessive
NKX2-2	NM_002509	Neonatal diabetes and developmental delay	Recessive
PAX6	NM_001604	Aniridia and impaired glucose tolerance	Dominant
PCBD1	NM_000281	Hyperphenylalaninemia and diabetes	Recessive
PDX1	NM_000209	Permanent neonatal diabetes +/- pancreatic agenesis	Recessive
		MODY	Dominant
PLIN1	NM_002666	Familial Partial Lipodystrophy (FPLD4) and insulin resistance	Dominant
POLD1	NM_002691	Mandibular hypoplasia, deafness, progeroid features, and lipodystrophy (MDPL) syndrome	Dominant (de novo)
PPARG	NM_015869	Familial Partial Lipodystrophy (FPLD3) and insulin resistance	Dominant
PTF1A	NM_178161	Permanent neonatal diabetes with cerebellar and pancreatic agenesis	Recessive
RFX6	NM_173560	Permanent neonatal diabetes with pancreatic hypoplasia, intestinal atresia, and gallbladder aplasia or hypoplasia	Recessive
		MODY	Dominant
SIRT1	NM_012238	Monogenic autoimmune diabetes	Dominant
SLC2A2	NM_000340	Fanconi-Bickel syndrome	Recessive

SLC19A2	NM_006996	Thiamine responsive megaloblastic anaemia, diabetes and deafness (TRMA) syndrome	Recessive
SLC29A3	NM_018344	H syndrome & PHID syndrome	Recessive
STAT1	NM_007315	Immunodeficiency 31C and IPEX-like phenotype	Dominant
STAT3	NM_139276	Neonatal diabetes and poly-autoimmune disease	Dominant/Recessive
STAT5B	NM_012448	Growth hormone insensitivity with immunodeficiency	Recessive
TRMT10A	NM_001134665	Juvenile-onset diabetes with microcephaly, epilepsy and intellectual disability	Recessive
WFS1	NM_006005	Wolfram syndrome (Diabetes insipidus, diabetes mellitus, optic atrophy and deafness, DIDMOAD)	Recessive
ZFP57	NM_001109809	Transient neonatal diabetes	Recessive

ESM Table 4. Genetic etiology and clinical characteristics of monogenic diabetes patients identified in this study

study id	Gene	Variant	Protein effect	ACMG classification	Genotype	Sex	Age at diagnosis (years)	BMI percentile	Duration of diabetes	Parents with diabetes	Consanguineous parents	HbA1c (%/mmol/mol	T1D-GRS	T1D-GRS centile	GADA	ICA	IAA	IA-2A	Treatment at recruitment	Additional reported features
11	*GCK*	NM_000162.3:c.1222G>T	p.Val408Leu	Likely pathogenic (4)	Heterozygous	Female	16.1	33	0.0	Yes	No	6/42	0.235	6	Neg	Neg	Neg		None	
30	*GCK*	NM_000162.3:c.127C>T	p.Arg43Cys	Pathogenic (5)	Heterozygous	Male	6.8	74	2.2	No	No	5.8/40	0.232	5	Neg	Neg	Neg		None	Hypothyroidism
51	*GCK*	NM_000162.3:c.683C>T	p.Thr228Met	Pathogenic (5)	Heterozygous	Male	4.9	33	3.2	Yes	Yes	6.4/46	0.266	30	Neg				OHA	
52	*GCK*	NM_000162.3:c.943C>T	p.Leu315Phe	Likely pathogenic (4)	Heterozygous	Female	10.6	1	0.1		No	6.5/48	0.235	5	Neg	Neg	Neg		None	
54	*INS*	NM_001185098.1:c.188-37T>A	p.?	Likely pathogenic (4)	Heterozygous	Female	1.6	77	3.0	No	No	7.1/54	0.245	10	Neg				Insulin	
55	*PTF1A*	NM_178161.2:c.571C>A	p.Pro191Thr	Pathogenic (5)	Homozygous	Female	7.7	87	9.4	No	Yes	11.2/99	0.218	1					Insulin	Coeliac disease, cataracts, skeletal dysplasia, mental retardation
75	*SLC19A2*	NM_006996.2:c.237C>A	p.Tyr79*	Pathogenic (5)	Homozygous	Male	3.5	84	5.1	No	No	8/64	0.273	40	Neg	Neg	Neg		Insulin	Deafness, megoblastic anemia
93	*HNF1A*	NM_000545.6:c.872dup	p.Gly292Argfs*25	Pathogenic (5)	Heterozygous	Female	10.1	88	4.4	Yes	No	9/75	0.235	6	Neg	Neg			Insulin	
117	*HNF1A*	NM_000545.6:c.723C>A	p.Cys241*	Pathogenic (5)	Heterozygous	Female	11.2	17	0.1	Yes	No	7/53	0.179	0	Neg	Neg	Neg		None	
118	*SLC19A2*	NM_006996.2:c.567_568insT	p.Leu190Serfs*51	Pathogenic (5)	Homozygous	Female	3.1	57	3.5	No	No	6.8/51	0.263	27	Neg	Neg			Insulin	Deafness, anemia, congenital heart disease (cardiomyopathy, ASD, TY), diabetes insipidus
131	*MT-RNR2*	NC_012920.1:n.MT-RNR2:*14A>G	m3243	Pathogenic (5)	Heteroplasmic	Female	6.1	41	7.6	No	No	8.4/68	0.166	0	Pos	Neg	Neg		Insulin	
160	*SLC19A2*	NM_006996.2:c.1265T>C	p.Leu422Pro	Likely pathogenic (4)	Homozygous	Male	4.1	60	0.1	No	Yes	6.8/51	0.242	9	Neg	Neg	Neg		Insulin	Hypermetropia,

																				astigmatism, anaemia (transfused, etiology unknown)
202	*HNF1A*	NM_000545.6:c.1400C>T	p.Pro467Leu	Pathogenic (5)	Heterozygous	Female	6.7	5	0.1	No	No	13.4/123	0.271	37	Neg	Neg	Neg		Insulin	
204	*HNF1B*	Deletion		Pathogenic (5)	Heterozygous	Male	12.9	59	0.1	Yes	No	14.6/136	0.268	34	Neg	Neg	Neg		Insulin	Autism
216	*INS*	NM_001185098.1:c.287G>C	p.Cys96Ser	Pathogenic (5)	Heterozygous	Male	2.0	26	12.7	No	No	14/130	0.232	4					Insulin	
217	*GCK*	NM_000162.3:c.943C>T	p.Leu315Phe	Likely pathogenic (4)	Heterozygous	Male	5.3	11	2.8	No	No	6.2/44	0.210	1	Neg	Neg	Neg		None	Bilateral inguinal hernia, bilateral undescended testis, premature birth (maternal pre-ecalmpsia)
239	*WFS1*	NM_006005.3:c.1919_1928del	p.Leu640Profs*15	Pathogenic (5)	Homozygous	Female	3.9	99	13.7	No	No	9.5/80	0.227	3	Neg	Neg	Neg		Insulin	Blindness and scoliosis (operated for it)
446	*GCK*	NM_000162.3:c.313del	p.His105Thrfs*11	Pathogenic (5)	Heterozygous	Male	6.2	2	1.9	No	Yes	6/42	0.218	1					None	
476	*GCK*	NM_000162.3:c.130G>A	p.Gly44Ser	Pathogenic (5)	Heterozygous	Male	6.8	70	8.1	Yes	No	6.9/52	0.238	7	Neg	Neg	Neg		OHA	
487	*WFS1*	NM_006005.3:c.529C>A	p.Arg177Ser	Likely pathogenic (4)	Homozygous	Male	4.9	99	4.7	Yes	No	11/97	0.181	0	Neg		Neg		Insulin	Deafness
541	*HNF4A*	NM_175914.4:c.278G>A	p.Cys93Tyr	Pathogenic (5)	Heterozygous	Female	13.9	98	2.6	No	No	5.8/40	0.222	2	Neg	Neg	Neg		OHA	
572	*GCK*	NM_000162.3:c.1174C>G	p.Arg392Gly	Pathogenic (5)	Heterozygous	Male	2.4	88	0.4	Yes	No	6.2/44	0.216	1		Neg	Neg		None	
597	*HNF1B*	NM_000458.3:c.443C>T	p.Ser148Leu	Pathogenic (5)	Heterozygous	Male	10.7	2	1.2	No	Yes	6.9/52	0.220	2					Insulin	
599	*WFS1*	NM_006005.3:c.1523_1524del	p.Tyr508Cysfs*34	Pathogenic (5)	Homozygous	Female	7.3	23	9.2	No	Yes	10.7/93	0.223	2	Neg			Neg	Insulin	optic atrophy, deafness, diabetes insipidus
701	*WFS1*	NM_006005.3:c.1215T>A	p.Tyr405*	Pathogenic (5)	Homozygous	Female	6.2	24	5.6	No	Yes	8.5/69	0.207	1	Neg			Neg	Insulin	Deafness

750	*SLC29A3*	NM_018344.5:c.607T>C	p.Ser203Pro	Likely pathogenic (4)	Homozygous	Male	6.5	63	4.0	No	Yes	8.8/73	0.243	10	Neg				Insulin	Deafness, vitiligo, short stuture
752	*KCNJ11*	NM_000525.3:c.481G>A	p.Ala161Thr	Likely pathogenic (4)	Heterozygous	Female	15.5	27	0.2	No	Yes		0.219	2					Insulin	Allergic asthma, imunnodeficiency
802	*WFS1*	NM_006005.3:c.776del	p.Ile259Thrfs*28	Pathogenic (5)	Homozygous	Male	3.4	36	3.3	No	Yes	8.8/73	0.239	7	Neg	Neg	Neg		Insulin	
838	*GCK*	NM_000162.3:c.645C>A	p.Tyr215*	Pathogenic (5)	Heterozygous	Male	14.5	32	4.4	Yes	No	6.1/43	0.266	30	Neg	Neg	Neg		OHA	
898	*WFS1*	NM_006005.3:c.1885C>T	p.Arg629Trp	Likely pathogenic (4)	Homozygous	Male	6.0	92	8.2	No	No	9.3/78	0.208	1	Neg	Neg			Insulin	constipation
908	WFS1	NM_006005.3:c.1232_1233del and NM_006005.3:c.2511G>A	p.Ser411Cysfs*131 and p.Trp837*	Pathogenic (5)	Compound Heterozygous	Female	6.7	13	3.7	No	No	8.5/69	0.239	7	Neg	Neg	Neg		Insulin	
1090	*GCK*	NM_000162.3:c.1222G>T	p.Val408Leu	Likely pathogenic (4)	Homozygous	Male	3.1	65	9.7	Yes	Yes	7.6/60	0.235	5	Neg				Insulin	
1241	*GCK*	NM_000162.3:c.1256del	p.Phe419Serfs*12	Pathogenic (5)	Heterozygous	Female	14.4	66	0.6	Yes	No	6.4/46	0.238	7	Neg	Neg	Neg		None	
1328	*TRMT10A*	NM_001134665.2:c.379C>T	p.Arg127*	Pathogenic (5)	Homozygous	Female	11.4	2	1.6	No	No	7.5/58	0.243	9	Neg	Neg	Neg		None	Growth hormone deficiency, mental retardation, microcephaly, deep set eyes

ESM Table 5: Characteristics of monogenic diabetes identified in our previous systematic study in the UK [1].

	Monogenic Diabetes, n=20	The rest of the cohort n=788	p
**Age at diagnosis (years)**	11 (8.5-14), 20	8 (4-11), 788	0.004
**Duration at recruitment (years)**	2.2 (0.6-3.2), 20	4.5 (1.7-8.2), 788	0.002
**BMI percentile**	89 (57-99), 16	79 (57-94), 776	0.2
**Female**	8/20 (40)	362/788 (46)	0.4
**White ethnicty**	19/20 (95)	773/788 (98)	0.3
**Parents with diabetes**	18/20 (90)	122/771 (16%)	<0.001
**Any antibody positive**	0/20 (0)	220/298 (74%)	<0.001
**C-peptide (pmol/l)**	2.5 (0.8-5.7), 16	0 (0-0.3), 784	<0.001
**HbA1c at recruitment, %**	7.3 (6.5-8.8), 16	8.5 (7.7-9.7), 777	0.004
**HbA1c at recruitment, mmol/mol**	56 (48-73), 16	69 (61-83), 777	
**HbA1c at recruitment ≤7.5%**	8/16 (50)	152/777 (20)	0.007
**Current treatment**			
**Non-insulin treated**	16/20 (80)	9/788 (1)	<0.001
**Insulin treated**	4/20 (20)	779/788 (99)	
**Insulin dose (units/kg/day)**	0 (0-0.3), 16	0.9 (0.7-1.1), 776	<0.001
**T1D-GRS**			
**MODY Prob ≥10%**	14/16 (88)	49/760 (6)	<0.001
**Genetic etiologies**	*GCK*, 8	N/A	
	HNF1A, 5		
	HNF4A, 4		
	HNF1B, 1		
	ABCC8, 1		
	INSR, 1		
**Autosomal dominant**	20/20 (100)		
**Autosomal recessive**	0/20 (0)		

Data are median (IQR) for continuous variable or number (%) for categorical variables. Number of people where the data was available is indicated after comma for continuous variables and after forward slash for categorical variables.

ESM Table 6. Genetic aetiologies of monogenic diabetes identified in routine referrals from the UK in patients with age at diabetes diagnosis between 0.5-20 y.

Genetic cause	Individuals (n)	Mode of inheritance	N individuals with white-European ethnicity	Individuals with other ethnicities	Comments
HNF1A	39	Dominant	33	6 (4 Southasian 1 Black, 1 Hispanic)	
HNF4A	19	Dominant	12	7 (3 Southasian , 1 Black, 1 Hispanic, 1 Arabic, 1 Mixed)	
GCK	12	Dominant	12	0	
HNF1B	8	Dominant	6	2 (1 Black, 1 Hispanic)	
RFX6	8	Dominant	7	1 (SA)	
INSR	5	Dominant	4	1 (Hispanic)	
GATA6	2	Dominant	2	0	
m.3243A>G	2	Mitochondrial	2	0	
ABCC8	2	Dominant	2	0	
ABCC8	1	Recessive	0	1 (Arabic)	Consanguineous parents
NEUROD1	1	Dominant	0	1 (SA)	
PPARG	1	Dominant	0	1 (Black)	
INS	1	Dominant	1	0	
STAT3	1	Recessive	0	1 (Arabic)	Consanguineous parents

ESM Table 7: Clinical characteristics of autosomal dominant and autosomal recessive monogenic diabetes, and the rest of the cohort.

	Monogenic diabetes autosomal dominant causes, n=19	Monogenic diabetes autosomal recessive causes, n=14	Rest of the cohort, n=1059	p Autosomal dominant cases vs the rest of cohort	p Autosomal recessive cases vs the rest of cohort	p Autosomal dominant vs autosomal recessive
**Age at diagnosis (years)**	10.1 (5.3-13.9), 19	5.4 (3.5-6.7), 14	8.2 (5.1-11.5), 1059	0.47	0.008	0.04
**Female**	9/19 (47)	7/14 (50)	530/1059 (50)	1.0	1.0	1
**Age at recruitment (years)**	11.9 (8.1-14.9), 19	11.1 (8.6-14.1), 14	12.5 (9.1-15.4), 1059	0.61	0.44	0.80
**BMI percentile**	33 (11-74), 19	61 (24-87), 14	61 (34-82), 1058	0.04	0.90	0.25
**Current treatment**						
**Non-Insulin**	12/19 (63)	1/14 (7)	17/1059 (2)	<0.001	0.21	0.001
**Insulin**	7/19 (37)	13/14 (93)	1042/1059 (98)			
**Insulin dose (units/kg/day)**	0.6 (0.4-0.7), 7	0.9 (0.8-1.2), 12	0.9 (0.7-1.1), 976	0.012	0.30	0.02
**Non-Autoimmune, extra-pancreatic features**	2/19 (11)	11/14 (79)	55/1043 (5)	0.27	<0.001	<0.001
**Parents with diabetes**	9/18 (50)	2/14 (14)	145/1043 (14)	<0.001	1.0	0.06
**Consanguineous parents**	4/19 (21)	7/14 (50)	212/1059 (20)	1	0.01	0.14
**HbA1c at recruitment, %**	6.5 (6.1-7.1), 18	8.7 (7.6-9.5), 14	8.7 (7.5-10.3), 1043	<0.001	0.79	0.01
**HbA1c at recruitment, mmol/mol**	48 (43-54), 18	72 (60-80), 14	72 (58-89), 1043			
**HbA1c ≤7.5% at recruitment**	14/18, (78)	3/14, (21)	264/1059 (25)	<0.001	1.00	0.005
**Islet autoantibody positive**	1/15 (0)	0/13 (0)	478/701 (68)	<0.001	<0.001	N/A
**Diabetes duration at time of antibody measurement, months**	0 (0-0), 14	41 (0-76), 11	0 (0-0), 656	0.66	<0.001	0.008
**C-peptide (pmol/l) within 6 months of diagnosis**	463 (226-804), 12	151 (77-430), 5	153 (83-278), 585	<0.001	0.92	0.09
**T1D-genetic risk score**	0.235 (0.219-0.245), 19	0.237 (0.218-0.243), 14	0.279 (0.258-0.298), 1059	<0.001	<0.001	0.97
**Composite clinical probability of MODY ≥10%**	9/12 (75)	2/13 (15)	65/890 (7)	<0.001	0.25	0.005

Data are median (IQR) for continuous variable or number (%) for categorical variables. Number of people where the data was available is indicated after comma for continuous variables and after forward slash for categorical variables. MODY, Maturity Onset Diabetes of the Young.

ESM Table 8. **Performance of patient selection criteria for identifying monogenic diabetes**. Odds ratio for each criteria is calculated against the rest of cohort (n=1059).

**Paediatric diabetes clinic cohort from Turkey - a population with high rates of consanguinity**				
	**Autosomal dominant cases, n=19**		**Autosomal recessive cases, n=14**	
**Patient selection criteria**	**Odds ratio (95% CI)**	**p**	**Odds ratio (95% CI)**	**p**
Parents with diabetes	6.2 (2.1-17.9)	0.0003	1 (0.1-4.7)	1
Non-insulin treatment	105.1 (32.6-347.9)	<0.0001	4.7 (0.1-34.9)	0.2
HbA1c ≤7.5% (58 mmol/mol)	10.3 (3.2-43.4)	<0.0001	0.8 (0.1-3.1)	1
Composite clinical probability of MODY ≥10%	38 (9.1-221.2)	<0.0001	2.3 (0.2-10.9)	0.3
Non-autoimmune extra pancreatic features	2.1 (0.2-9.4)	0.3	66.9 (16.8-379.1)	<0.0001
Consanguineous parents	1.1 (0.3-3.4)	1	4 (1.2-13.5)	0.012
**Paediatric diabetes clinic cohort from the UK**(2)**, predominantly nonconsanguineous population**				
	**Autosomal dominant cases, n=20**		**Autosomal recessive cases, n=0**	
	**Odds ratio (95% CI)**	**p**		
Parents with diabetes	47.9 (11.2-427.7)	<0.0001	-	-
Non-insulin treatment	346.1 (85-1680.8)	<0.0001	-	-
HbA1c ≤7.5% (58 mmol/mol)	4.1 (1.3-12.8)	0.0068	-	-
Composite clinical probability of MODY ≥10%	101.6 (22-929.9)	<0.0001	-	-

ESM Table 9: The list of extra-pancreatic non-autoimmune features that were reported by the clinicians at recruitment in patients with monogenic diabetes.

Cataracts
Anaemia
Autism
Bilateral inguinal hernia
Bilateral undescended testis
Blindness
Congenital heart disease
Deafness
Diabetes insipidus
Growth hormone deficiency
Hypermetropia
Developmental Delay
Microcephaly
Optic atrophy
Scoliosis
Short stature
Skeletal dysplasia
Constipation

ESM Table 10. Systematic review of Genetic Testing Registry for the monogenic diabetes gene panels. We undertook systematic search in Gene testing registry (https://www.ncbi.nlm.nih.gov/gtr/) on 12th June 2020 We used the following search criteria - Diabetes mellitus[DISNAME] AND (testtype_clinical[PROP]) AND ("Next-Generation (NGS)/Massively parallel sequencing (MPS)"[METHOD]) AND (testpurpose_diagnosis[PROP]), C0342276[DISCUI] OR C0342277[DISCUI], C3888631[DISCUI] to identify laboratories that offered gene panel tests for monogenic diabetes. We identified 26 laboratories that offered 34 gene panel tests for monogenic diabetes. MODY, Maturity onset diaberes of the young; NDM, Neonatal diabetes

Name of the genetic lab	Country	Type of panel offered	No. of genes on the panel	No. of recessive monogenic diabetes genes on the panel	Maximum no. of recessive genes in non-NDM panel	*WFS1* in Non-NDM panel	*SLC19A2* in Non-NDM panel
Al Jalila Children’s Genomics Center Al Jalila Childrens Speciality Hospital	United Arab Emirates	MODY	17	6	6	No	No
Ambry Genetics	United States	MODY	5	2	2	No	No
Arkansas Children's Hospital Arkansas Childrens Hospital	United States	MODY	11	5	5	No	No
Asper Biogene	Estonia	MODY	15	7	7	No	No
Blueprint Genetics	Finland	MODY, Comprehensive Monogenic Diabetes	13, 28	6, 13	6	Yes	No
CEN4GEN Institute for Genomics and Molecular Diagnostics	Canada	NDM	5	4	-	-	-
Centogene AG - the Rare Disease Company	Germany	MODY, NDM	15, 10	8, 6	8	No	No
CGC Genetics	Portugal	Hyperglycemia and diabetes, NDM	74, 13	25, 9	25	Yes	Yes
DDC Clinic Molecular Diagnostics Laboratory DDC Clinic, Center for Special Needs Children	United States	MODY, Monogenic diabertes	13, 26	5, 10	10	Yes	No
EGL Genetic Diagnostics Eurofins Clinical Diagnostics	United States	MODY	4	2	2	No	No
Esoterix LabCorp	United States	MODY	4	1	1	No	No
Fulgent Genetics	United States	MODY & Neonatal Diabetes	30	15	15	Yes	No
GeneDx	United States	MODY	16	5	5	No	No

GENETAQ Molecular Genetics Centre and Diagnosis of Rare Diseases	Spain	MODY, NDM	11, 17	3, 12	3	No	No
Genetic Diagnostic Laboratory University of Pennsylvania School of Medicine	United States	MODY	15	5	5	No	No
Genetic Services Laboratory University of Chicago	United States	MODY, MODY& NDM, NDM	14, 45,15	5, 22, 9	22	Yes	No
Greenwood Genetic Center Diagnostic Laboratories Greenwood Genetic Center	United States	MODY	14	5	5	No	No
Instituto de Medicina Genomica	Spain	MODY	4	1	1	No	No
Knight Diagnostic Laboratories - Molecular Diagnostic Center Oregon Health & Science University	United States	MODY	25	14	14	No	No
Laboratorio de Genetica Clinica SL	Spain	MODY & Diabetes Neonatal	25	14	14	No	No
Laboratory for Molecular Diagnostics Center for Nephrology and Metabolic Disorders	Germany	MODY	11	4	4	No	No
LifeLabs Genetics	Canada	MODY, Monogenic Diabetes	16, 43	8, 13	13	Yes	No
Molecular Genetics Laboratory - Diagnostics Genetics LabPLUS - Auckland City Hospital	New Zealand	MODY	4	1	1	No	No
PreventionGenetics	United States	MODY	14	6	6	No	No
Reference Laboratory Genetics	Spain	MODY, NDM	11, 16	4, 12	4	No	No
Synlab MVZ Humane Genetik München	Germany	MODY	7	3	3	No	No

ESM Fig. 1: Flow chart showing study recruitment and genetic testing pathway. Genetic etiologies and the number of affected individuals is indicated. T1D-GRS, Type 1 diabetes Genetic Risk score**.** All cases (n=111) with low T1D-GRS (<0.234,<5th centile of T1D population) and 125 individuals with negative islet autoantibodies and low to moderate T1D-GRS (0.234-0.280) (5th-50th centile of T1D population) were selected for genetic testing

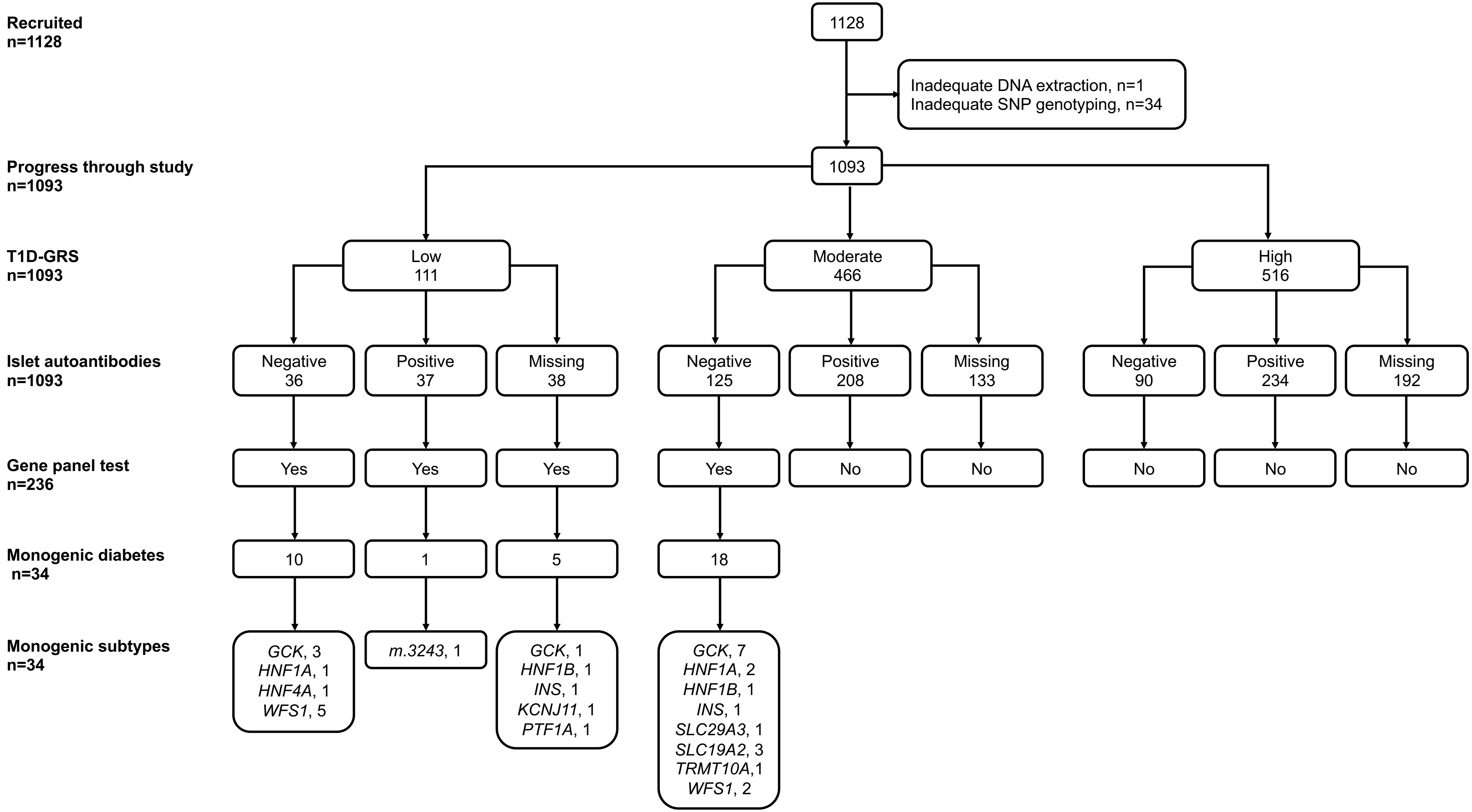

ESM Fig. 2. T1D-GRS of children with T1D from European and Turkish ancestry. *The graph showing T1D-GRS for the 1963 European T1D children from the WTCCC study and the 472 Turkish children with definite T1D (clinically diagnosed with t1D, insulin treated from diagnosis and positive for islet autoantibodies).* the Mann-Whitney U test *P*=0.48, two-sample Kolmogorov-Smirnov test *P*=0.37

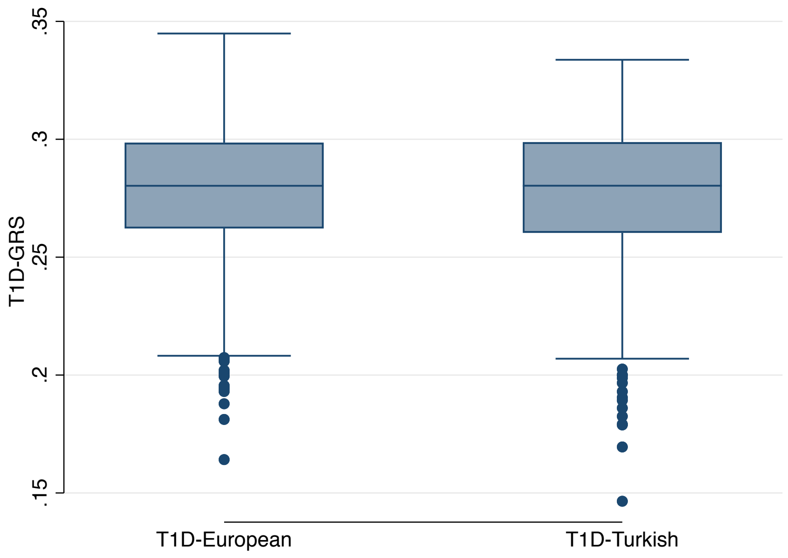

ESM Reference

[1] Shepherd M, Shields B, Hammersley S, et al. (2016) Systematic Population Screening, Using Biomarkers and Genetic Testing, Identifies 2.5% of the U.K. Pediatric Diabetes Population With Monogenic Diabetes. Diabetes Care 39(11): 1879-1888. 10.2337/dc16-0645

[2] Patel KA, Oram RA, Flanagan SE, et al. (2016) Type 1 Diabetes Genetic Risk Score: A Novel Tool to Discriminate Monogenic and Type 1 Diabetes. Diabetes 65(7): 2094-2099. 10.2337/db15-1690

[3] Ellard S, Lango Allen H, De Franco E, et al. (2013) Improved genetic testing for monogenic diabetes using targeted next-generation sequencing. Diabetologia 56(9): 1958-1963. 10.1007/s00125-013-2962-5

[4] Richards S, Aziz N, Bale S, et al. (2015) Standards and guidelines for the interpretation of sequence variants: a joint consensus recommendation of the American College of Medical Genetics and Genomics and the Association for Molecular Pathology. Genet Med 17(5): 405-424. 10.1038/gim.2015.30
